# Molecular Odor Prediction Using Olfactory Receptor Information

**DOI:** 10.1002/minf.202400274

**Published:** 2025-03-13

**Authors:** Yuta Wakutsu, Hiromasa Kaneko

**Affiliations:** ^1^ Department of Applied Chemistry School of Science and Technology Meiji University 1-1-1 Higashi-Mita, Tama-ku 214-8571 Kawasaki, Kanagawa Japan

**Keywords:** fragrance, molecular descriptor, protein descriptor, odor, olfactory receptor

## Abstract

In fragrance development, the framework development process is a bottleneck from the perspective of labor, cost, and human resource development. Odors vary greatly depending on the structure and functional groups of the molecule. Although odor has been predicted from only the structure of molecules, its practical application remains elusive. In this study, we developed a model for predicting the odor of molecules that have only small differences in structure. Focusing on the mechanism of human olfaction, we divided the mechanism into three levels and constructed three models: a classification model that predicts the presence or absence of binding between molecules and olfactory receptors, a regression model that predicts the strength of binding, and a classification model that predicts the presence or absence of odor based on the strength of binding. Olfactory receptors were used as descriptors to discriminate between similar molecular odors. Our models predicted odor differences between some similar molecules, including optical isomers.

## Graphical abstract

We developed models to predict the odors of structurally similar molecules by focusing on human olfaction mechanisms. These models differentiated odors between similar molecules, including optical isomers.

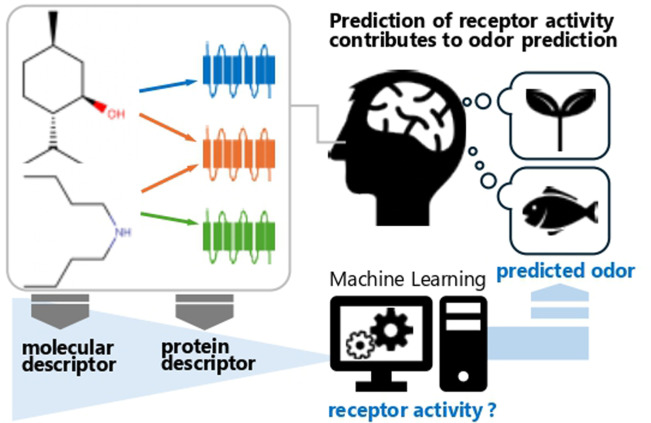

## 1 Introduction

Fragrance development involves planning, framework development, and optimization. Challenges in framework development include the labor and cost of trial and error, in which several perfumers spend several months handling a wide variety of raw materials [1].

Quantitative structure–activity relationship (QSAR) methods [2] can predict the correlation between the structural features of a molecule and its biological activity. By considering odor rather than activity, a QSAR model may be able to predict odor from the structure of a molecule. However, using a QSAR model to predict odor is difficult because: 1) olfaction is subjective, and even experts find it challenging to share information; 2) odor molecules activate multiple olfactory receptors with various affinities; and 3) small differences in molecular structure or functional groups can significantly change odor [3]. In an international crowd‐sourced competition, some teams built models that successfully predicted odor from molecular structure alone; however, these models have not been put to practical use because of these and other difficulties [4].

Menthol varies in odor with slight structural differences. Specifically, d‐menthol and l‐menthol were odorally distinguishable, with l‐menthol having a significantly stronger minty odor than d‐menthol [5]. Because of their structural similarity, it is difficult to predict odor differences between two optical isomers from the molecular structure and functional groups alone. However, the mouse olfactory receptors MOR161‐2 and MOR171‐16 were found to be specific for l‐menthol and d‐menthol, respectively [6]. Thus, the interaction between olfactory receptors and molecules may be useful in odor prediction.

The aim of this study was to build an odor prediction model that accounts for small structural differences by incorporating both olfactory receptor and molecular structure information, focusing on the fact that odors are highly variable due to small differences in molecular structure and functional groups – a challenge in predicting odor using machine learning methods.

Relationships between molecules and olfactory receptors related to odor recognition mechanisms have been reported [7]. Examples of these relationships are shown in Table 1, which was created using data from the dataset and previous studies described in Section 2.1. Molecules that bind to the same receptor have the same odor, whereas molecules that bind to different receptors have different odors. Generally, the odor of a molecule is determined by whether it binds to olfactory receptors and the strength of the binding. In this study, the odor prediction process was as follows: Step 1, prediction of binding to olfactory receptor; Step 2, prediction of binding strength; and Step 3, prediction of odor from combination of binding and binding strength.

**Table 1:** Examples of the relationships between olfactory receptors and odors.

Molecule	Odor	MOR271‐1	OR2W1	OR10J5
3‐Heptanone	Sweet	○	○	×
3‐Octanone	Sweet	○	○	×
Lyral	Woody	×	×	○

Therefore, we constructed three models corresponding to each step, namely Models 1, 2, and 3, respectively. To validate these models, we checked whether they discriminated odor differences using molecules other than those used to construct the model. The models were also used to predict the odor of new molecules.

The benefits of using odor prediction models in flavor and fragrance development include: 1) reducing development time and cost by eliminating formulations that do not need to be tried, thereby limiting the scope of the search; 2) expanding the scope by suggesting formulations that may not have been thought of [1]. Among machine learning studies related to the human senses, olfaction has lagged behind vision and hearing, including face recognition and automatic speech recognition [8, 9, 10], mainly because of the difficulties listed above. In this study, we exploited the interaction between molecules and olfactory receptors, to help advance research in the still developing field of olfaction.

## 2 Computational Methods

### 2.1 Dataset

We used data from a previous study [11] for 63 different molecules for 62 different mouse or human olfactory receptors. The data were given as the logarithm of the half maximal effective concentration (log(EC50) [12], which is the concentration at which a molecule or compound shows 50% of its maximum response. Three model datasets were generated from these data. The molecules’ odors were obtained by searching OlfactionBase [13]. We found cases where molecules were reported to bind to receptors in previous studies, but were listed as not binding in OlfactionBase; these receptors were excluded from the model datasets. Thus, 61 olfactory receptors were used in this study.

The dataset for Model 1 contained 3843 samples, all of which are combinations of molecules and olfactory receptors. Samples with log(EC50)=0 were considered negative and samples with log(EC50) values >0 were considered positive.

The dataset for Model 2 contained only the samples that were considered positive in the Model 1 dataset. The objective variable was log(EC50), and the number of samples was 333.

To create the dataset for Model 3, we first searched OlfactionBase for the odors of the 63 molecules in the previous study [11]. This time, we obtained odor data for 56 molecules with a total of 23 odors. However, because olfaction is subjective, each molecule can have multiple representations of an odor. Therefore, we grouped odors with strong relationships that are easily judged as close odors. We categorized the odors based on graph theory. The connections between odors were represented as a graph using NetworkX [14], a graph network handling library, then community extraction was performed. The extraction method was based on Clauset‐Newman‐Moore greedy modularity maximization [15]. Community extraction is an approach that extracts dense parts of a network without predefining the number of divisions. Starting from an initial state where all vertices have separate community labels, each vertex repeatedly changes its community affiliation to match the surrounding vertices. Modularity, expressed by the following equation, is used as an indicator to measure the quality of the community:

(1) $$\begin{array}{c} Q=\frac{1}{2m}\sum_{ij}\left(A_{ij}-\frac{k_{i}k_{j}}{2m}\right)\delta \left(C_{i},\;C_{j}\right) \end{array}$$

where *m* is the total number of edges in the network, *A_ij_* is the (*i*, *j*) element of the adjacency matrix *A* (1 if vertices *i* and *j* are connected by an edge, otherwise 0), *k_i_* and *k_j_* are the degrees of vertices *i* and *j* respectively, and *δ*(*C_i_*, *C_j_*) is a function that is 1 if vertices *i* and *j* are in the same community, otherwise 0. The 23 odors were divided into six communities as Odors 1–6. The characteristics of these communities are described in Section 3.3. The Mode 3 dataset contained the combined information on whether the 56 molecules exhibited one of the six odors and the log(EC50) values of the 56 molecules for the 61 different olfactory receptors.

### 2.2 Descriptors

Molecular and protein descriptors were used in this study., The molecular descriptors include the physical and chemical properties for 200 molecules calculated using RDKit [16] (RDKit descriptors), Morgan fingerprints (Morgan, 2048 bits, radius of 2) [17], RDKit fingerprints (RDKit) [18], and MACCS Keys fingerprints (MACCS Keys) [19].

The protein descriptors include amino acid frequency (AAF) [20] and descriptors for amino acid type and position (alignment) [21]. For AAF, we used the number of amino acids counted one by one (1AAF), the number of adjacent amino acids (2AAF), the number of amino acids in a sequence of three (3AAF), and the number converted to a percentage (1AAFperc, 2AAFperc, and 3AAFperc, respectively). In a previous study [21], amino acid sequences were converted into a two‐dimensional matrix of amino acid types and positions for alignment. In this study, we used the two‐dimensional matrix as a variable by rearranging it into one dimension. For multiple sequences, it is necessary to align the lengths of the sequences, and therefore we used multiple alignment for this purpose.

Alignment is the process of comparing multiple sequences and extracting and organizing common regions to determine the similarity between sequences. Gaps are inserted into multiple amino acid sequences so that identical or similar amino acids are vertically aligned [22]. The structure and function of the sequences can be inferred from the relationships between the compared sequences by vertically aligning similar parts.

### 2.3 Modelling Methods

The classification methods considered in Models 1 and 3 were logistic regression analysis [23], linear discriminant analysis [24], naive Bayes classifier, k‐nearest neighbor algorithm, linear support vector machine [25], non‐linear support vector machine [25, 26], decision tree [27], random forests (RF) [28], light gradient boosting machine (LGBM) [29], eXtreme gradient boosting (XGB) [30], and gradient boosting decision tree (GBDT) [27].

The regression analysis methods considered in Model 2 were partial least squares regression [31], ridge regression [32], least absolute shrinkage and selection operator [33], elastic net [34], linear support vector regression [35], non‐linear support vector regression (NLSVR) [35], RF [28], LGBM [29], XGB [30], decision tree [27], ordinary least squares [36], GBDT [27], and gaussian process regression (GPR) [37].

**Figure 1:**
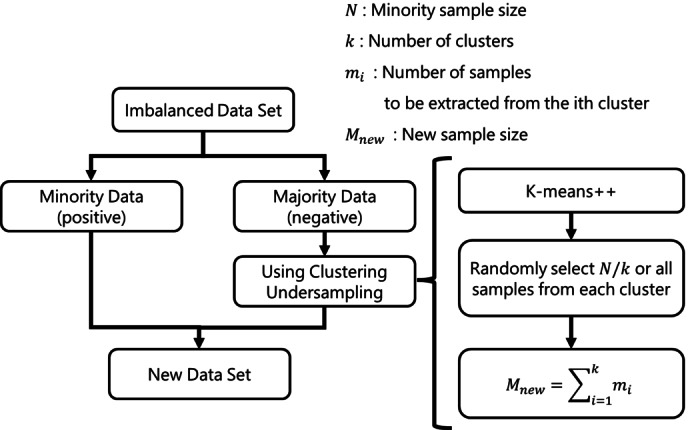
Overview of the clustering undersampling method used in this study.

In terms of hyperparameter tuning, among classification methods, the linear support vector machine was optimized for “C” using cross‐validation. The non‐linear support vector machine was optimized for “C” and “gamma” using grid search cross‐validation. For regression methods, partial least squares regression was optimized for the number of components, while ridge regression and the least absolute shrinkage and selection operator (LASSO) were optimized for “alpha” using cross‐validation. The elastic net was optimized for “alpha” and “L1 ratio,” the linear support vector regression was optimized for “C” and “epsilon,” and the non‐linear support vector regression was optimized for “C,” “epsilon,” and “gamma” using grid search cross‐validation. In all cases, the number of folds was set to 5. The default values were used as the hyperparameters in the other classification and regression methods. Classification methods often fail to identify molecules and olfactory receptors that bind to each other, resulting in more negative samples than positive samples. Therefore, for Models 1 and 3, we used clustering as an undersampling method [38] to reduce the number of samples for the majority group and maintain balance in the number of samples. An overview of the undersampling method is shown in Figure 1. The k‐means++ clustering algorithm was used as the clustering method [39].

### 2.4 Metrics

The metrics for the classification models, Models 1 and 3, were accuracy, precision, recall, and F‐measure. The four evaluation indices were calculated as follows:

(2) $$\begin{array}{c} \mathrm{Accuracy}\;=\frac{TP+TN}{TP+FP+N+TN} \end{array}$$

(3) $$\begin{array}{c} \mathrm{Precision}\;=\frac{TP}{TP+FP} \end{array}$$

(4) $$\begin{array}{c} \mathrm{Recall}\;=\frac{TP}{TP+FN} \end{array}$$

where *TP* is true positive, *TN* is true negative, *FP* is false positive, and *FN* is false negative. The closer the indices are to 1, the better the performance of the classification model.

The metrics for the regression model, Model 2, were the coefficient of determination (r2), the mean absolute error (MAE), and the root mean square error (RMSE). The three metrics were calculated as follows:

(6) $$\begin{array}{c} r^{2}=1-\frac{\sum_{i=1}^{n}{\left(y^{\left(i\right)}-y_{EST}^{\left(i\right)}\right)}^{2}}{\sum_{i=1}^{n}{\left(y^{\left(i\right)}-y_{mean}\right)}^{2}} \end{array}$$

(7) $$\begin{array}{c} \mathrm{MAE}=\frac{\sum_{i=1}^{n}\left|y^{\left(i\right)}-y_{EST}^{\left(i\right)}\right|}{n} \end{array}$$

(8) $$\begin{array}{c} \mathrm{RMSE}=\sqrt{\frac{\sum_{i=1}^{n}{\left(y^{\left(i\right)}-y_{EST}^{\left(i\right)}\right)}^{2}}{n}} \end{array}$$

where $y^{\left(i\right)}$ is the value of the objective variable for the *i*‐th sample, $y_{EST}^{\left(i\right)}$ is the estimated value of the objective variable for the *i*‐th sample, *y*_mean_ is the mean of the objective variable, and *n* is the number of samples.

### 2.5 Flow of Odor Prediction

**Figure 2:**
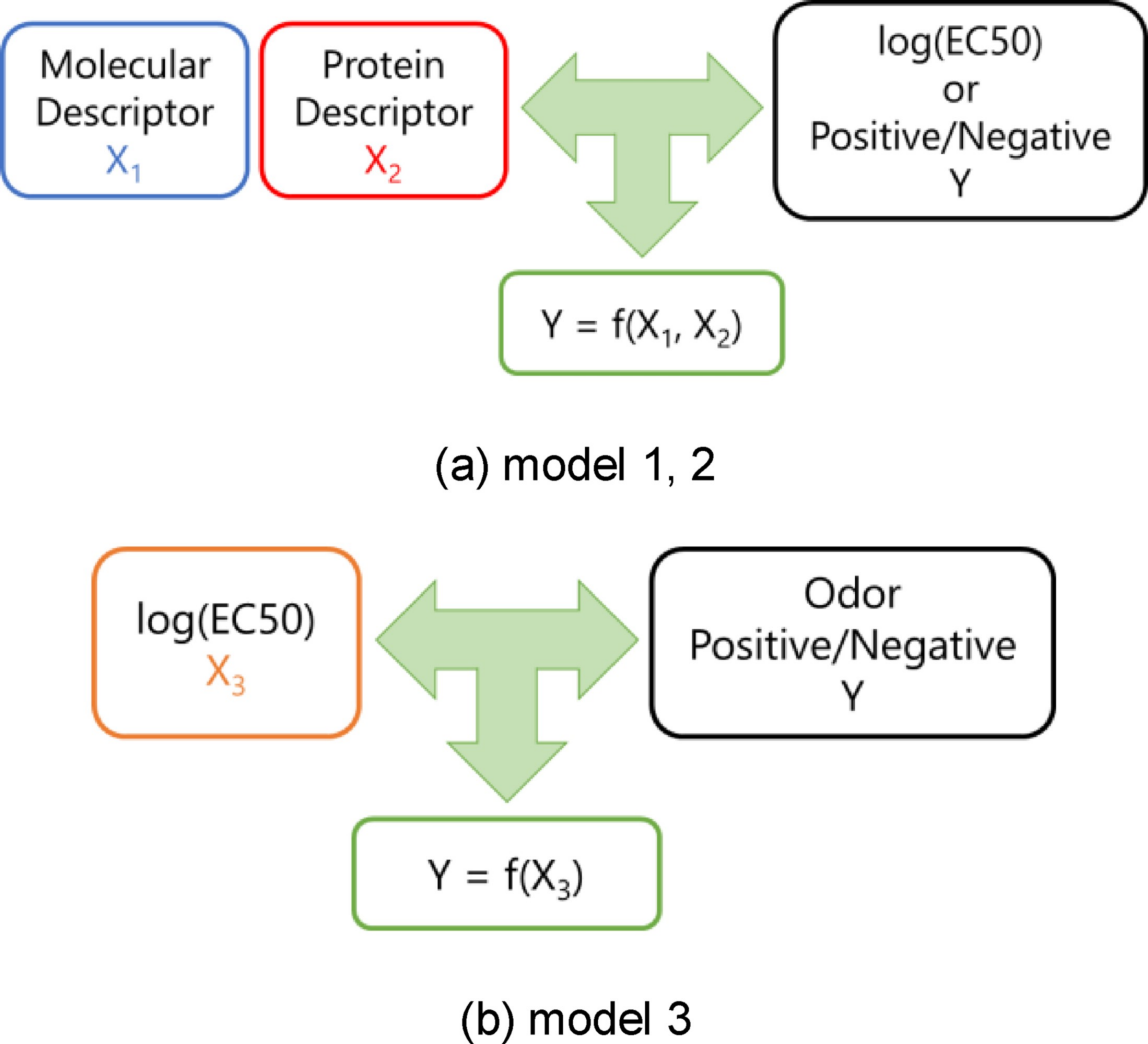
Conceptual diagram of model construction. (a) Model 1 is a classification model in which the objective variable is positive or negative, indicating whether the molecule binds to the olfactory receptor; Model 2 is a regression model in which the objective variable is log(EC50), indicating the strength of binding of the molecule to the olfactory receptor. The explanatory variables are the molecular descriptor X_1_ and the protein descriptor X_2_, which provide information on the olfactory receptor. The specifics of the descriptors are described in Section 2.2. (b) Model 3 is a class classification model in which the objective variable is positive or negative indicating whether the molecule has or does not have an odorant community; the explanatory variable is log(EC50) for each receptor. log(EC50), logarithm of the half maximal effective concentration.

A schematic diagram of the predictive models used in this study is shown in Figure 2. To predict the presence or absence of six different communities, Model 3 was divided into six two‐class classification models.

Because the sample size is small (56 samples), we used double cross‐validation or nested cross‐validation to validate the models. Nested cross‐validation is a method used to separate hyperparameter tuning and model evaluation. By using this method, overfitting can be prevented, and the performance of the model can be evaluated more accurately. First, the dataset is divided into multiple folds, and outer cross‐validation is performed. For each fold, inner cross‐validation is conducted using the remaining folds to find the optimal hyperparameters. The model is then evaluated on the outer test set using the found hyperparameters. This process is repeated for all folds to evaluate the final model performance.

This method ensures that hyperparameter selection does not influence the test set evaluation, allowing for a more accurate measurement of the generalization performance of the model. Nested cross‐validation is particularly effective when the dataset has a small sample size. This is because it maximizes the use of limited data and allows for a more accurate evaluation of the performance of the model. With a small sample size, simply splitting the data into training and test sets can result in even smaller data amounts for each set, leading to unstable model evaluations. By using nested cross‐validation, the entire dataset is used multiple times for both training and testing, efficiently utilizing all available data.

As described in the introduction, the odor prediction is done in three steps using the three models.

In Step 1, Model 1 is used to predict the activity between a molecule for which odor is unknown and the 61 olfactory receptors used to construct the model. Because the odor of only one molecule is to be predicted and there are 61 olfactory receptors, there are 61 molecule–receptor combinations to be considered. By inputting these combinations into Model 1, binding and non‐binding olfactory receptors can be identified.

In Step 2, Model 2 is used to predict the log(EC50) of the bound molecule–receptor combinations. Only combinations that were predicted to bind by Model 1 are entered into Model 2. The log(EC50) of the combinations predicted not to bind is set to 0. Thus, Models 1 and 2 predict the log(EC50) of molecules for which the odor is unknown that are predicted to bind to 61 different olfactory receptors.

In Step 3, Model 3 is used to predict the odor communities of the molecules for which the odor is unknown odor by entering the log(EC50) for the 61 olfactory receptors into the six two‐class classification models.

## 3 Results and Discussion

### 3.1 Model 1

We combined a set of molecular descriptors and a set of protein descriptors, then built models with different patterns and compared the prediction accuracy among the combinations. The results for the F‐measure are shown in Table 2. The results for the accuracy, precision, and recall are shown in Tables S1–S3, respectively.

**Table 2:** F‐measure for best classification result for each molecule–receptor combination.

	RDKit descriptor		RDKit		Morgan		MACCS Keys	
1AAF	XGB	0.831	LGBM	0.829	RF	0.832	LGBM	0.834
1AAFperc	RF	0.850	LGBM	0.837	RF	0.841	LGBM	0.824
2AAF	XGB	0.846	LGBM	0.844	LGBM	0.826	LGBM	0.837
2AAFperc	LGBM	0.836	XGB	0.818	LGBM	0.828	RF	0.815
3AAF	RF	0.860	LGBM	0.825	LGBM	0.864	XGB	0.843
3AAFperc	LGBM	0.838	LGBM	0.825	LGBM	0.844	XGB	0.824
alignment	LGBM	0.858	LGBM	0.814	LGBM	0.837	XGB	0.830

AAF, amino acid frequency; XGB, eXtreme gradient boosting; RF, random forest; LGBM, light gradient boosting machine.

The best descriptor combination was Morgan+3AAF (Table 2). 3AAF tended to be more accurate not only for Morgan, but also for the RDKit descriptors and MACCS Keys. 2AAF had higher values for three of the four molecular descriptors compared with the values for 1AAF and 2AAF. Therefore, the accuracy tended to be higher when the number of consecutive amino acids to be counted was higher, probably because it better reflects the characteristics of the receptor. Comparing 1AAF, 2AAF, and 3AAF with 1AAFperc, 2AAFperc, and 3AAFperc did not necessarily increase accuracy. Therefore, the accuracy seems to depend on which of the descriptors, the number of amino acids or the ratio, is better for improving accuracy.

Additionally, models were constructed by combining two types of protein descriptors and by concatenating all molecular and protein descriptors. However, in both cases, the accuracy of the models did not improve. Table S4 shows the results of the two combinations with the highest accuracy and the results when all descriptors were used. Due to the large number of variables, variable selection was performed using Boruta [40].

### 3.2 Model 2

#### 3.2.1 Comparison of the Distinction Between Mouse and Human Receptors

**Figure 3:**
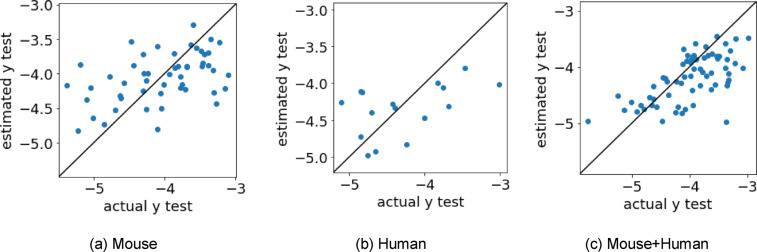
Scatter plots of the log(EC50) values estimated by the best method (dataset) and measured log(EC50) values. log(EC50), logarithm of the half maximal effective concentration.

The data used in this study included both mouse and human receptors from a previous study [11]. Therefore, we constructed prediction models for mouse and human separately and together, and compared their prediction accuracy. The evaluation indices for each model are shown in Table 3, and scatter plots of the log(EC50) values estimated by the best method and the measured log(EC50) values are shown in Figure 3. The dataset with the highest accuracy was mouse+human (Table 3), and more samples were located near the diagonal line with the mouse+human dataset (Figure 3). The high accuracy may be because the number of samples was increased by combining the two datasets. The validity of combining the two datasets suggests that the relationship between the EC50 of the molecule and receptor is similar for the mouse and human receptors. The combined mouse+human dataset was used in the subsequent regression model building.

**Table 3:** Prediction accuracy of models constructed using different datasets or molecular descriptors.

	Mouse	Human	Mouse Human	Mouse Human	Mouse Human	Mouse Human	Mouse Human
Molecular	RDKit	RDKit	RDKit	RDKit	RDKit	Morgan	MACCS
descriptor	descriptor	descriptor	descriptor	descriptor			keys
Best method	LGBM	RR	LGBM	LGBM	RF	LGBM	NLSVR
r^2^	0.209	0.220	0.266	0.266	0.247	0.290	0.380
RMSE	0.542	0.512	0.484	0.484	0.490	0.476	0.445
MAE	0.435	0.430	0.373	0.373	0.388	0.374	0.348

1AAF was used as the protein descriptor. LGBM, light gradient boosting machine; RR, ridge regression; RF, random forest; NLSVR, non‐linear support vector regression.

#### 3.2.2 Comparison of Molecular Descriptors

**Figure 4:**
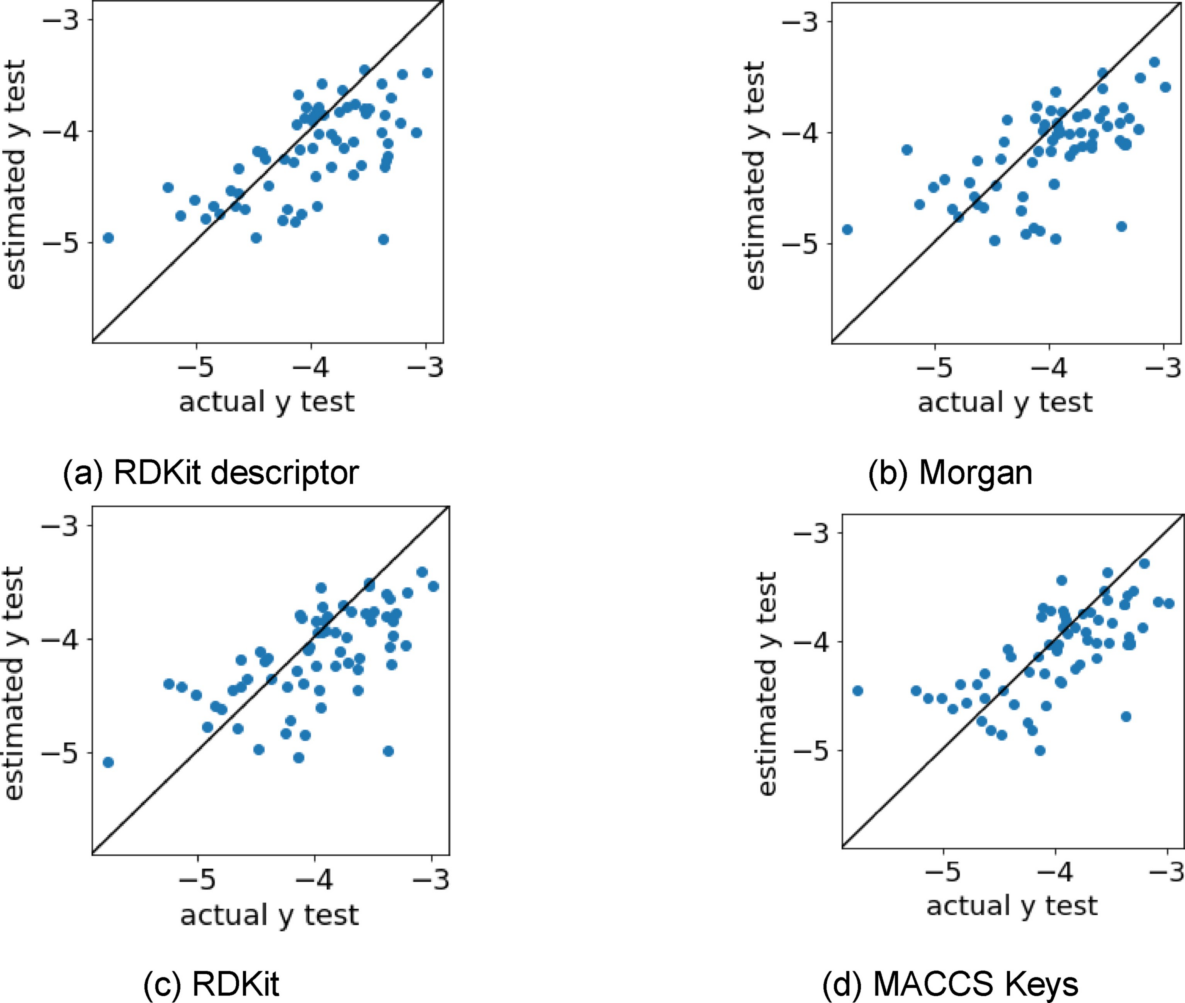
Scatter plots of the log(EC50) values estimated by the best method (molecular descriptor) and measured log(EC50) values. log(EC50), logarithm of the half maximal effective concentration.

Models were built using RDKit descriptors, Morgan fingerprints, RDKit fingerprints, and MACCS Keys fingerprints, and their accuracies were compared. 1AAF was used as the protein descriptor. The evaluation indices for each molecular descriptor are shown in Table 3, and scatter plots of the log(EC50) values estimated by the best method and the measured log(EC50) values are shown in Figure 4. MACCS Keys was the most accurate molecular descriptor (Table 3), and MACCS Keys had a relatively large number of samples near the diagonal line (Figure 4). Because MACCS Keys is a molecular descriptor that indicates the presence or absence of 166 substructures, the molecular substructures in this dataset are considered important for prediction. MACCS Keys was used as the molecular descriptor in the subsequent regression model building.

#### 3.2.3 Comparison of Protein Descriptors

**Figure 5:**
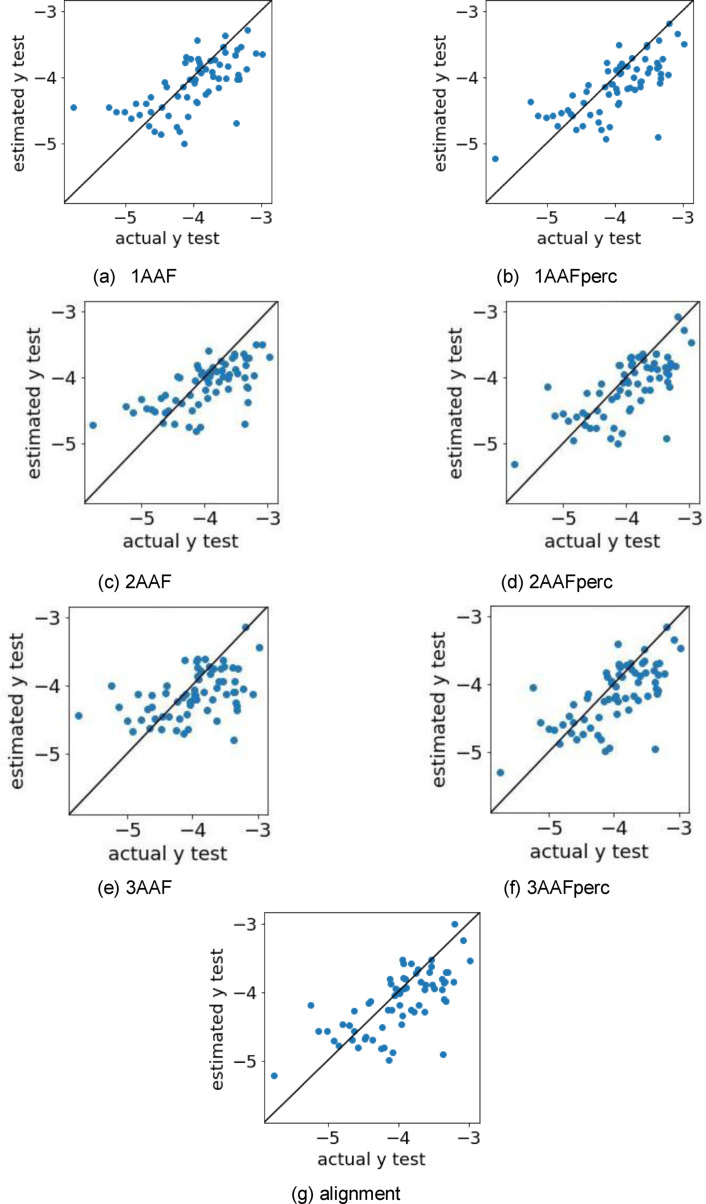
Scatter plots of the log(EC50) values estimated by the best method (protein descriptor) and measured log(EC50) values. AAF, amino acid frequency; log(EC50), logarithm of the half maximal effective concentration.

Models were constructed using five descriptors, 1AAF, 3AAF, 1AAFperc, 3AAFperc, and alignment, and their accuracies were compared. 1AAF and 1AAF consider the number of amino acids, and 3AAF, 3AAFperc, and alignment consider the order of amino acids. The evaluation indices of each protein descriptor are shown in Table 4, and scatter plots of the log(EC50) values estimated by the best method and the measured log(EC50) value are shown in Figure 5. The results show that 1AAFperc was the most accurate protein descriptor.

**Table 4:** Evaluation metrics for each protein descriptor.

	1AAF	2AAF	3AAF	1AAFperc	2AAFperc	3AAFperc	alignment
Best method	LGBM	NLSVR	LGBM	LGBM	LGBM	LGBM	LGBM
r^2^	0.367	0.373	0.369	0.438	0.381	0.347	0.377
RMSE	0.450	0.447	0.449	0.424	0.445	0.457	0.446
MAE	0.344	0.345	0.349	0.328	0.345	0.350	0.349

MACCS Keys was used as the molecular descriptor. AAF, amino acid frequency; LGBM, light gradient boosting machine; NLSVR, non‐linear support vector regression. r^2^, coefficient of determination; RMSE, root mean square error; MAE, mean absolute error.

By converting the number of amino acids to a percentage and comparing 1AAF and 1AAFperc and 3AAF and 3AAFperc, we found that the accuracy increased for 1AAFperc and decreased for 3AAFperc (Table 4). For 1AAFperc, the accuracy was considered to have improved because the length of the amino acid sequence can be considered by converting the AAF to a ratio. For 3AAFperc, which considers the order of amino acids, the ratio was not effective.

Alignment was the best descriptor when comparing 3AAF, 3AAF perc, and alignment, as shown in Table 4. 3AAF and 3AAFperc consider the order of three amino acids, whereas alignment considers the order of all the amino acids in a sequence, and therefore can be more effective in considering the order of all amino acids. However, the accuracy of alignment was relatively different from that of 1AAFperc, implying that the proportion of amino acids was a better descriptor than the order of amino acids. 1AAF was also shown to be better than the method of counting by separating two or more amino acids in the original paper on AAF [20], and therefore this was considered to be a reasonable result.

Additionally, models were constructed by combining two types of protein descriptors and by concatenating all molecular and protein descriptors. Table S5 shows the evaluation metrics for each combination, and Figure S1 shows a scatter plot with the estimated values from the best method on the vertical axis and the actual values on the horizontal axis. The combination of 1AAF perc + 3AAF + alignment had the highest accuracy, surpassing the best 1AAF perc in the 3‐2‐3 method. It is considered that 3AAF and alignment supplemented the information that could not be represented by 1AAF perc.

### 3.3 Model 3

The results of the six odor communities analysis are shown in Table 5. Relatively close odors such as tobacco smoke and industrial odors in Odor 2 and sweet and caramel in Odor 6 tended to be grouped together. Within Odor 1, there are “Characteristic” and “Other”. While these notes are too abstract to be considered as odor notes, it is interesting to understand what kind of smell people associate with these terms, considering that some molecules in OlfactionBase are labeled as “Characteristic” and “Other” while others are not. Although “Characteristic” and “Other” are not sufficiently specific for describing odors and can be considered for exclusion to improve the accuracy of future models, we used “Characteristic” and “Other” as odor notes. The exploration of odor notes that provide qualitative olfactory information and the exclusion of overly abstract notes are important [41], which is an element for future consideration in our study and should continue to be discussed as a common issue among researchers in the field of olfaction.

**Table 5:** Results of the six odor communities analysis.

Community	Odors
Odor 1	Sulphur/Cabbage/Garlic, Marshy/Septic/Sulfurous, Characteristic, Other, Bakery, Medicinal/Alcohol, Microbial, Fuel/Gas Station/Solvent, Balsamic, Solvent/Hydrocarbon, Nature, Pungent, Beverage, Sickening, Foul, Chemical/Hydrocarbon
Odor 2	Tobacco smoke, Nose Feel, Rancid, Microbiological, Herbaceous, Industrial odors, Fragrant/Fruity, Sharp/Pungent, Earthy/Musty/Moldy, Dry woods, Nauseating, Fecal/Sewery, Earthy
Odor 3	Fishy/Ammonia, Popcorn, Burnt, Waste, Non‐vegetarian, Cleaning materials, Nutty, Fishy/Rancid, Vegetables, Floral, Citrus, Waxy, Green, Ammonia, Plants
Odor 4	Clean, Fresh, Fruity, Rancid/Putrid, Non‐food items, Ripe/Unripe, Chlorinous/Ozonous, Vegetation, Water, Food, Woody, Grassy/Woody, Building materials & construction
Odor 5	Terpenes/Pine/Lemon, Lemon, Fragrant, Medicinal/Phenolic, Chocolate, Non‐citrus fruity, Estery, Minty, Soft oriental, Brown, Metallic, Soft floral, Boiled
Odor 6	Sweet, Woody/Resinous, Restaurant, Spices, Caramel, Dry fruit, Dry, Aromatic, Oriental, Dairy

When converting molecular odors to odor communities, some odor communities were divided into majority and minority odor communities depending on the type of odor. If the odor communities of a molecule were unbalanced, we considered it better to interpret the molecule as having only one majority odor community. Therefore, we focused only on those odor communities that accounted for more than 20% of the total number of odor communities of a molecule. For example, we considered it more reasonable to classify a molecule with nine odors classified as Odor 1 and one odor classified as Odor 2 as having only Odor 1 than to classify it as having Odor 1 and Odor 2. A total of six models were constructed to predict the presence or absence of these odor communities.

**Figure 6:**
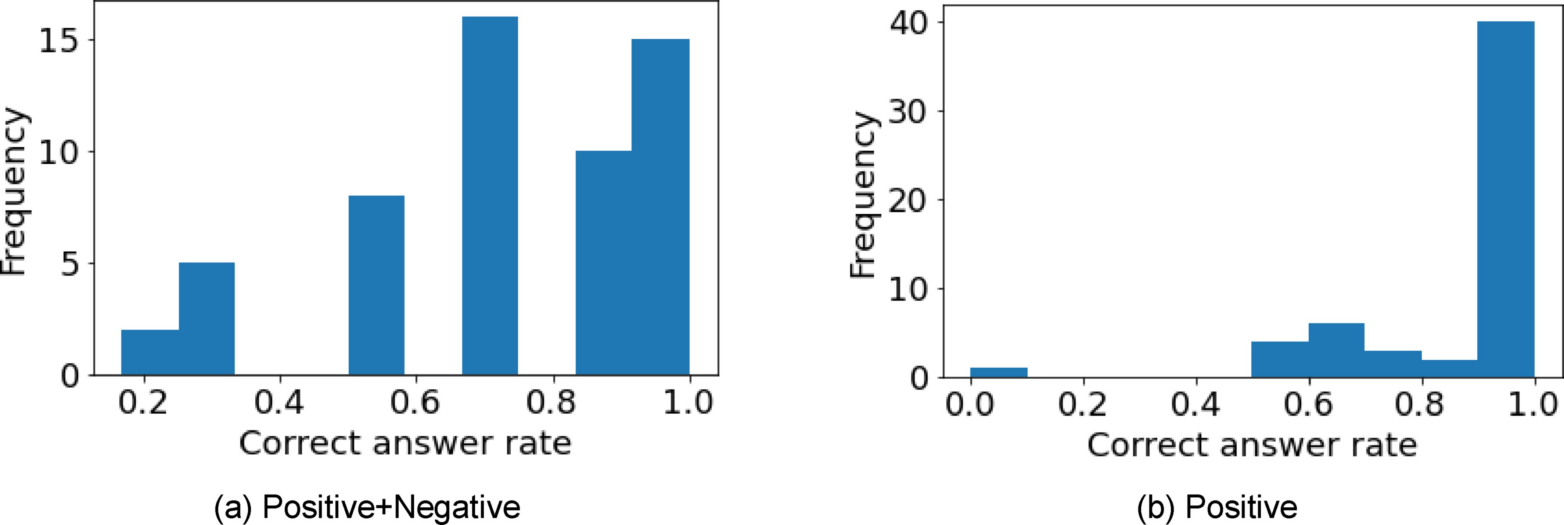
Correct answer rate for each molecule.

The metrics of the classification results for each model are shown in Table 6. The results show that relatively high accuracy was achieved with a small number of samples. Next, the accuracy for each molecule was calculated by dividing the sum of the six values by 6, where 1 is the agreement between the measured and estimated values for the presence/absence of each odor community in the numerator and 0 is the disagreement. A histogram of the accuracy for each numerator is shown in Figure 6, where (a) is the percentage of correct responses considering both presence and absence of odor, and (b) is the percentage of correct responses considering only the actual positive odor. More than half of the molecules had >50% correct answers (Figure 6a), and most of the time the correct answer is given when the odor is predicted to be present (Figure 6b). Although all six models had >70% correct rates (Table 6), some molecules had <30% correct rates (Figure 6), suggesting that some features of the molecules were not learnt by the models.

**Table 6:** Evaluation metrics for each odor community.

Odor	Best Method	Accuracy	Precision	Recall	F‐measure
1	DT	0.789	0.773	0.850	0.810
2	LSVM	0.871	0.810	1.00	0.895
3	LSVM	0.875	0.870	0.909	0.889
4	LSVM	0.729	0.720	0.750	0.735
5	LR	0.875	0.923	0.800	0.857
6	LSVM	0.838	0.815	0.957	0.880

DT, decision tree; LSVM, linear support vector machine; LR, logistic regression analysis.

### 3.4 Validation of the Models

To verify the odor estimation performance of the three constructed models, we tested them with molecules that were not used in the model construction. We used the models to estimate the odor of molecules with known odors and compared the estimates with the actual odors. We used 30 samples of molecules from previous studies [3, 10, 42] for validation, and manually converted their odors to odor communities using the method described in Section 3.3 for comparison.

**Figure 7:**
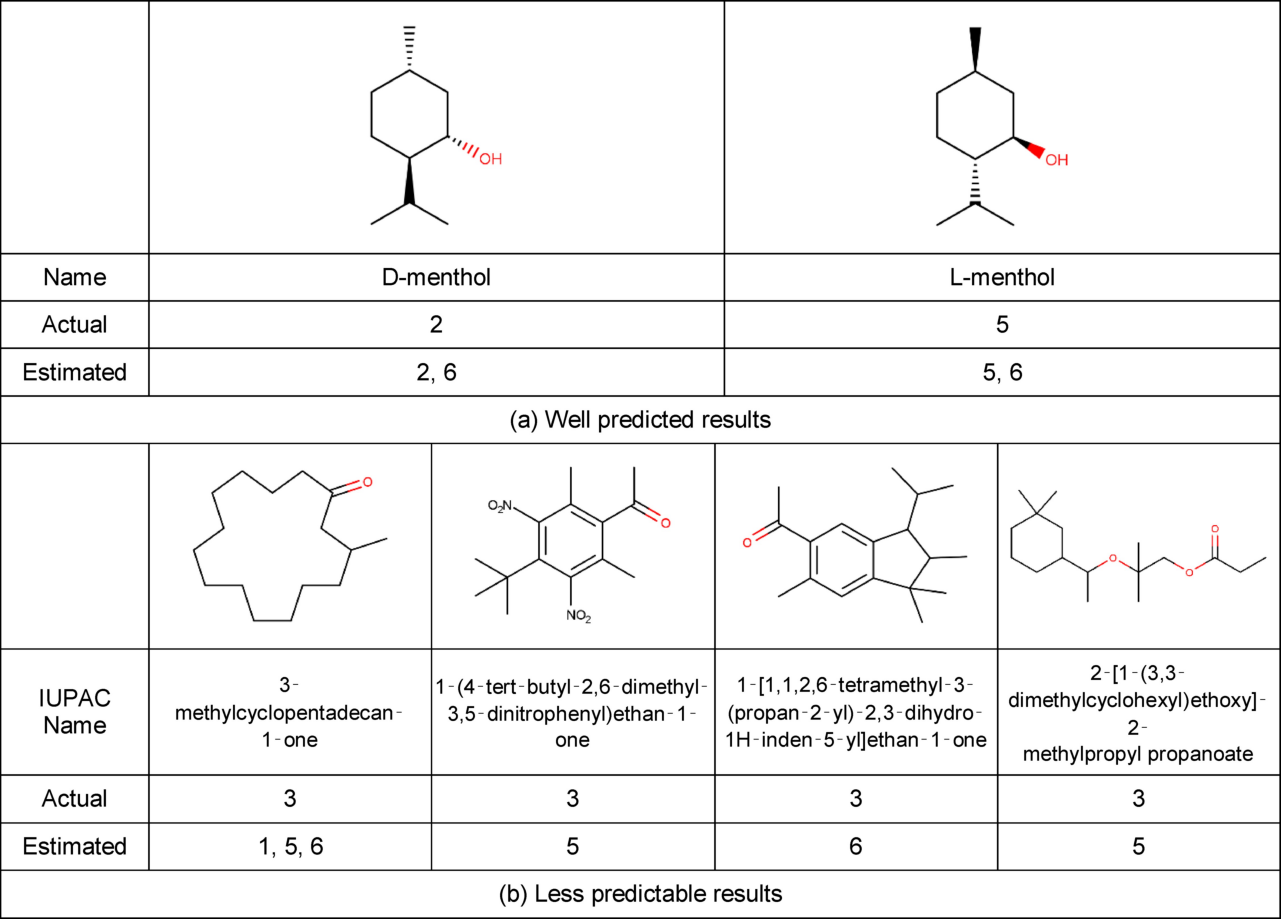
Excerpt of comparison between estimated and actual odor communities. Numbers indicate the number of the odor community.

The comparisons of the estimated and actual odors produced both well and poorly predicted results (Figure 7). A software MarvinView [43] was used to visualize chemical structures. The results for optical isomers of menthol show that despite being optical isomers, the difference in odor was accurately distinguished (Figure 7a). (Menthol was not included in the molecules used to construct the model.) Therefore, the odor differences of the optical isomers of menthol were discriminated by models built using the information of molecules other than menthol. MOR161‐2 and MOR‐171‐16, to which d‐menthol and l‐menthol specifically bind, were not included in the model construction. Therefore, it can be said that the odor was predicted from information other than the actual binding receptor. Given the report from FlavorBase Leffingwell [44], it is indeed possible that d‐menthol has a weak minty note and therefore could be predicted to have both Odor5 and Odor 2. As a future direction to improve the accuracy of our prediction model, a mechanism that can account for the intensity of the odors that molecules possess should be incorporated.

Model 1 had the ability to distinguish between enantiomers due to the use of the Morgan fingerprint. The calculation of the Morgan fingerprint utilized isomeric SMILES, which consider stereochemistry. By calculating the Morgan fingerprint based on isomeric SMILES, the stereochemical information of the enantiomers could be considered. In fact, the binding predictions for 61 receptors between the enantiomers of menthol differed in Model 1.

A group of molecules with different structures but the same odor was not accurately predicted (Figure 7b). To improve accuracy, more molecules need to be used in the model construction and more needs to be known about the relationship between the molecule structures and the olfactory receptors for odor prediction. Additional comparisons are shown in Figure S2.

**Figure 8:**
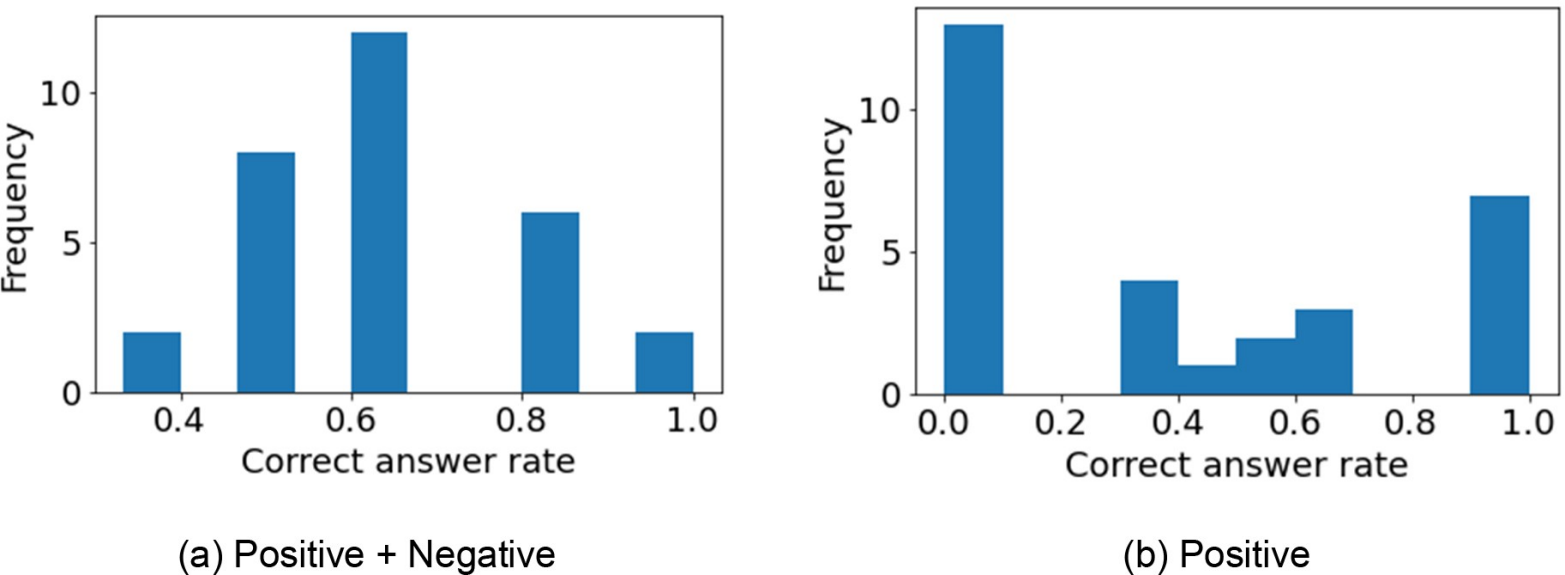
Correct answer rate for each molecule for verification.

A histogram of the accuracy for each molecule tested is shown in Figure 8. By comparing Figure 8a and Figure 8b, we found that the positive+negative molecules had higher accuracy, indicating that the negative contribution to accuracy was high. However, although the odor of some optical isomers was identified, the accuracy of odor prediction was not high. The names and odors of the validation molecules are shown in Table S6.

### 3.5 Prediction of Large‐Scale Data

To confirm the benefits and future challenges of the proposed method for fragrance development, odor prediction was performed on large‐scale data. We obtained 499,724 molecules from the Namiki Shoji database [45]. The Prediction was performed using only organic molecules but excluding organometallic complexes. The final number of extracted molecules was 354,180. Table 7 shows the number of predicted molecules for the six odor communities. The predicted molecules are realistic and purchasable, and many of them are not currently used as fragrances. Utilizing existing molecules in fragrance development is an effective strategy because it eliminates the need to explore synthetic routes and makes it easier to comply with environmental regulations. This result indicates the possibility that there are many molecules that can be utilized in fragrances and that the proposed method can contribute to narrowing down promising molecules. On the other hand, the applicability domain of the model was not specified in the prediction. Model 3, which predicts odor communities, requires a log(EC50) value for each of the 61 olfactory receptors. Therefore, if even one molecule–receptor pair in Model 1 or Model 2 is judged to be outside the applicability domain and therefore excluded from the prediction, the information necessary for the Model 3 prediction will be missing. A method for introducing the applicability domain needs to be established before the reliability of the prediction accuracy can be assessed.

**Table 7:** Odor reverse analysis.

Odor	Number of molecules obtained
1	199534
2	36535
3	29723
4	137453
5	100717
6	88886

## 4 Conclusions

In this study, the biological process of odor recognition was used to predict odor based on the presence and strength of the binding between a molecule and its olfactory receptors. By not relying solely on the structure of the molecule, our models discriminated odor differences between similar molecules, including optical isomers.

To address the issue of individual differences in odor perception, we used graph theory to detect connections between odors and successfully summarized highly related odors even when a molecule had many odors.

There would be three possible reasons for the unsatisfactory results. First, the number of odors contained within each odor community might have affected the performance of the model. Since we grouped 23 types of odors into 6 odor communities, there are some combinations of odors that seem out of place. It can be said that the number of odors contained within an odor community influences the prediction accuracy. It has been pointed out that due to the subjectivity of odors, there is often a lack of consistent specific terminology for odors, and the same odor can be described differently by different people. Previous studies have shown that different odors can induce the same emotions [46]. The communities presented in this study would encompass words that indicate the same odor or odors that induce similar emotions. In the future, it will be necessary to perform various processes, including the integration or deletion of labels, in the labeling of molecular odors. Additionally, when constructing Model 3 for each of the 23 odors, the number of molecules corresponding to each odor becomes smaller, leading to significant data imbalance in model construction. This could potentially degrade the accuracy of the model, which we recognize as a future challenge. Second, due to the structure of the proposed method, it is not possible to set an Applicability Domain. Some of the molecules predicted for validation in Section 3–7 may not have reliable predictions, which we recognize as a future challenge. Third, the diversity of molecular structures used for model construction is a factor. We used 63 molecules, which may not have been sufficient to predict the validation molecules accurately.

Considering these factors, a practical approach to improving prediction results would be to expand the data used for model construction. Increasing the variety of molecules would allow the model to handle a wider range of chemical structures. Expanding the types of olfactory receptors can enable the model to more flexibly capture the relationships between odors and receptors. Because obtaining the log(EC50) for all combinations of molecules and olfactory receptors experimentally is necessary, using predicted values as an alternative should also be considered.

In future research developments following the proposed model, we aim not only to improve prediction accuracy but also to link it to the interpretation of the relationship between molecules and odors. Although neural network models [47] have high prediction performance, the models are difficult to interpret. Our proposed method uses olfactory receptor information, which allows us to analyze the prediction results from a biological perspective. Our model will help elucidate the largely unexplored relationship between molecules and odors. By improving the predictive accuracy of our model, the reliability of the interpretations of molecular‐odor relationships proposed by our model can be ensured.

## Supporting Information

Additional supporting information can be found online in the Supporting Information section.

5

## Supporting information

As a service to our authors and readers, this journal provides supporting information supplied by the authors. Such materials are peer reviewed and may be re‐organized for online delivery, but are not copy‐edited or typeset. Technical support issues arising from supporting information (other than missing files) should be addressed to the authors.

Supporting Information

## Data Availability

The data that support the findings of this study are available in OlfactionBase at https://olfab.iiita.ac.in/olfactionbase/, reference number 13. These data were derived from the following resources available in the public domain: – odors, https://olfab.iiita.ac.in/olfactionbase/
