# Supplementary material for: Molecular Odor Prediction Using Olfactory Receptor Information
Source: Mol Inform. 2025 Mar 13;44(3):e202400274. doi: 10.1002/minf.202400274 (PMC11906144; doi:10.1002/minf.202400274)
Supplement: Supplementary file 1 — Supporting Information [file MINF-44-e202400274-s001.pdf]

Molecular Informatics

Supporting Information

Molecular Odor Prediction Using Olfactory Receptor Information

Yuta Wakutsu | Hiromasa Kaneko

Supplementary Data

Title: Molecular odor prediction using olfactory receptor information

Authors: Yuta Wakutsu[a] and Hiromasa Kaneko*[a]

Table S1. Accuracy for best classification result for each molecule–receptor combination.

	RDKit descriptor		RDKit		Morgan		MACCS Keys	
1AAF	XGB	0.821	LGBM	0.823	RF	0.821	RF	0.823
1AAFperc	RF	0.838	LGBM	0.827	RF	0.838	LGBM	0.812
2AAF	XGB	0.836	LGBM	0.838	LGBM	0.817	LGBM	0.827
2AAFperc	XGB	0.823	LGBM	0.812	LGBM	0.817	RF	0.806
3AAF	RF	0.853	LGBM	0.818	LGBM	0.856	XGB	0.833
3AAFperc	LGBM	0.826	LGBM	0.815	LGBM	0.839	XGB	0.812
alignment	RF	0.848	LGBM	0.806	LGBM	0.829	RF	0.818

AAF, amino acid frequency; XGB, eXtreme gradient boosting; RF, random forest; LGBM, light gradient boosting machine.

Table S2. Precision for best classification result for each molecule–receptor combination.

	RDKit descriptor		RDKit		Morgan		MACCS Keys	
1AAF	XGB	0.804	LGBM	0.819	RF	0.801	RF	0.814
1AAFperc	XGB	0.819	XGB	0.813	RF	0.843	RF	0.800
2AAF	XGB	0.818	LGBM	0.826	RF	0.809	LGBM	0.805
2AAFperc	XGB	0.797	LGBM	0.808	RF	0.805	RF	0.798
3AAF	NSVM	0.839	LGBM	0.813	RF	0.840	NSVM	0.823
3AAFperc	LGBM	0.797	LGBM	0.798	LGBM	0.836	LGBM	0.792
alignment	RF	0.829	RF	0.799	LGBM	0.815	RF	0.810

AAF, amino acid frequency; LGBM, light gradient boosting machine; RF, random forest; NSVM, non-linear support vector machine; XGB, eXtreme gradient boosting.

Table S3. Recall for best classification result for each molecule–receptor combination.

	RDKit descriptor		RDKit		Morgan		MACCS Keys	
1AAF	RF	0.882	LGBM	0.841	RF	0.865	LGBM	0.868
1AAFperc	RF	0.897	LGBM	0.871	LGBM	0.844	LGBM	0.859
2AAF	RF	0.885	XGB	0.885	LGBM	0.853	XGB	0.876
2AAFperc	LGBM	0.882	XGB	0.849	LGBM	0.862	LGBM	0.832
3AAF	RF	0.885	RF	0.853	LGBM	0.891	XGB	0.874
3AAFperc	LGBM	0.885	LGBM	0.856	LGBM	0.853	XGB	0.862
alignment	RF	0.885	XGB	0.838	LGBM	0.859	XGB	0.879

AAF, amino acid frequency; XGB, eXtreme gradient boosting; RF, random forest; LGBM, light gradient boosting machine.

Table S4. Evaluation metrics for classification results for Morgan+1AAFperc+3AAF and all descriptors.

	Morgan+1AAFperc+3AAF	All descriptors
Best method	RF	LGBM
Boruta *p*-percentile	45	55
Accuracy	0.868	0.832
Precision	0.863	0.798
Recall	0.882	0.900
F-measure	0.872	0.845

AAF, amino acid frequency; RF, random forest; LGBM, light gradient boosting machine.

Table S5. Evaluation metrics for each combination of protein descriptors.

Protein Descriptor	Best Method	r^2	RMSE	MAE	Boruta *p*-percentile
1AAFperc + 2AAFperc	LGBM	0.376	0.446	0.340	5
1AAFperc + 3AAF	NLSVR	0.428	0.427	0.327	60
1AAFperc + alignment	NLSVR	0.447	0.420	0.325	40
1AAFperc + 2AAFperc + 3AAF	NLSVR	0.405	0.436	0.338	5
1AAFperc + 3AAF + alignment	NLSVR	0.460	0.415	0.320	30
1AAFperc + 2AAFperc + alignment	LGBM	0.390	0.441	0.342	5
All descriptors	NLSVR	0.381	0.444	0.334	10

MACCS Keys was the molecular descriptor. AAF, amino acid frequency; LGBM, light gradient boosting machine; NLSVR, non-linear support vector regression; r2, coefficient of determination; RMSE, root mean square error; MAE, mean absolute error.

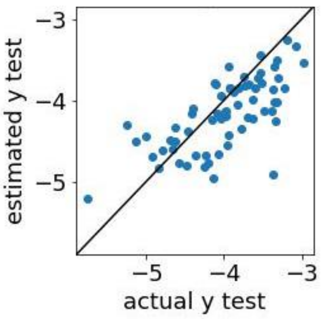

(a) 1AAFperc + 2AAFperc

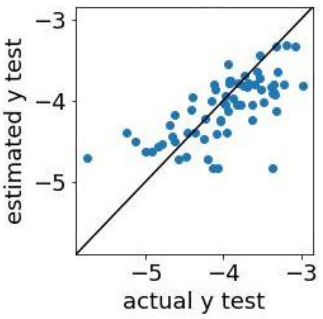

(b) 1AAFperc + 3AAF

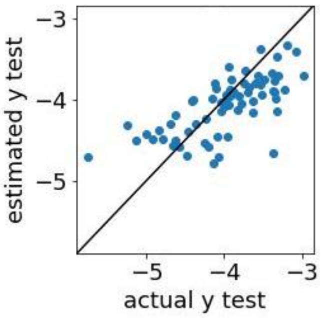

(c) 1AAFperc + alignment

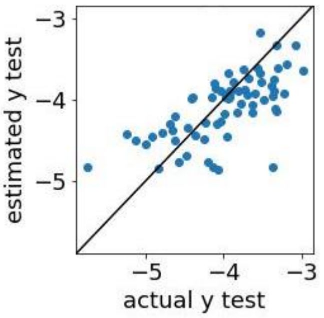

(d) 1AAFperc + 2AAFperc + 3AAF

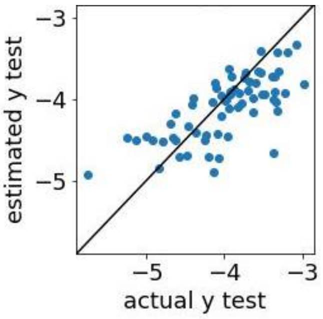

(e) 1AAFperc + 3AAF + alignment

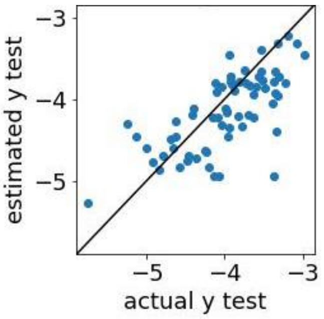

(f) 1AAFperc + 2AAFperc + alignment

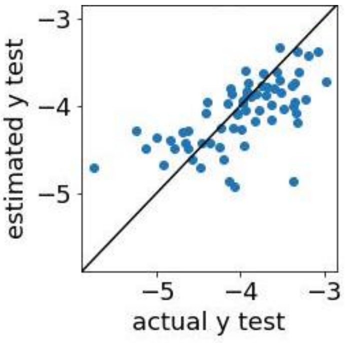

(g) All descriptor

Figure S1. Scatter plots of log(EC50) values estimated by the best method (combination) and measured log(EC50) values. AAF, amino acid frequency; log(EC50), logarithm of the half maximal effective concentration.

	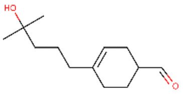	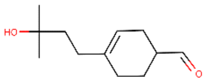	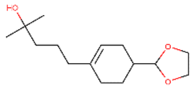	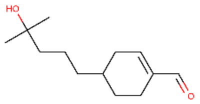	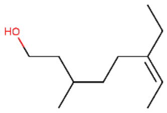
Actual	3, 4, 6	3	3, 4, 6	Odorless	3, 4, 5
Estimated	2, 4, 6	4, 5, 6	4, 5, 6	4, 5, 6	4, 5, 6

(a) Similar/different compounds of Lyral

	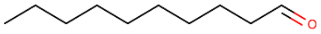	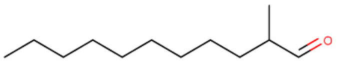	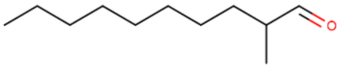
Actual	1, 3, 6	1, 3, 4, 5, 6	3, 4, 6
Estimated	3	3. 4	2, 3, 5, 6

(b) Same functional group, same odor (aldehyde)

	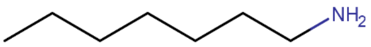	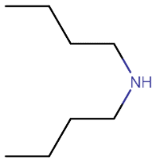
Actual	1, 3, 4	2, 3
Estimated	3	3

(c) Same functional group, same odor (amine)

	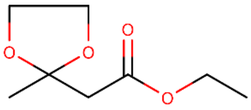	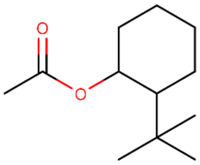	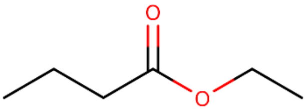
Actual	3, 4, 6	3, 4, 5	4, 6
Estimated	2, 4, 6	5	Odorless

(d) Same functional group, same odor (ester)

	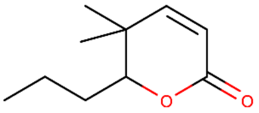	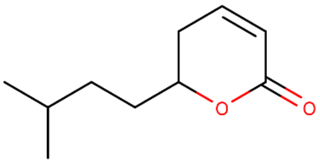	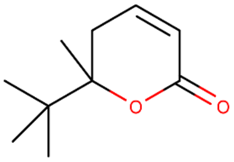
Actual	5	6	5
Estimated	5	5, 6	5

(e) Same functional group, different odor

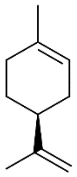 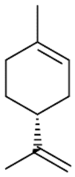

Actual	5	3
Estimated	1, 5, 6	1, 5

(f) Optical isomer 1

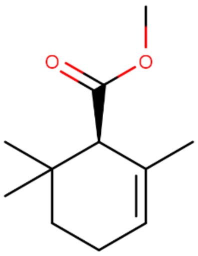

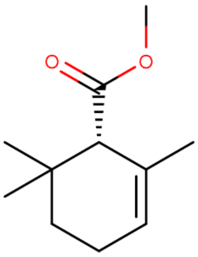

Actual	5	4
Estimated	5, 6	Odorless

(g) Optical isomer 2

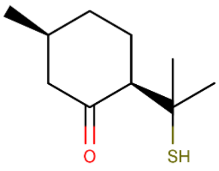

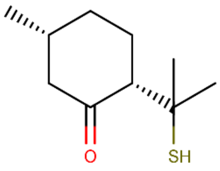

Actual	4	1
Estimated	5	5, 6

(h) Optical isomer 3

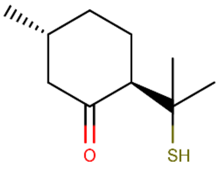

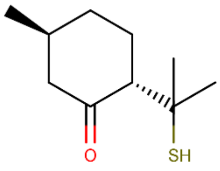

Actual	3	1, 4
Estimated	5, 6	5, 6

(i) Optical isomer 4

	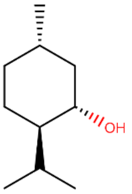	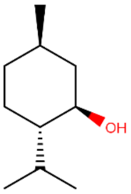
Actual	2	5
Estimated	2, 6	5, 6

(j) Optical isomer 5 (menthol)

	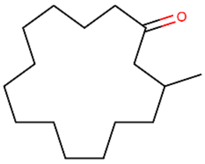	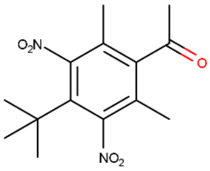	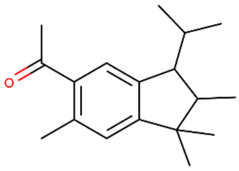	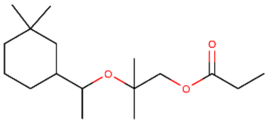
Actual	3	3	3	3
Estimated	1, 5, 6	5	6	5

(k) Different structure, same odor

Figure S2. Comparisons of estimated and actual odor communities. Numbers indicate the number of the odor community (see Table 5 in the main manuscript).

Table S6. Names and odors of molecules for verification.

Group	IUPAC Name	Other Name	Odor
a	4-(4-hydroxy-4-methylpentyl)cyclohex-3-ene-1-carbaldehyde	Lyral	Floral, Fresh, Sweet
	4-(3-hydroxy-3-methylbutyl)cyclohex-3-ene-1-carbaldehyde	-	Floral
	5-[4-(1,3-dioxolan-2-yl)cyclohex-1-en-1-yl]-2-methylpentan-2-ol	-	Floral, Fresh, Sweet
	4-(4-hydroxy-4-methylpentyl)cyclohex-1-ene-1-carbaldehyde	-	Odorless
	(6Z)-6-ethyl-3-methyloct-6-en-1-ol	-	Floral, Fresh, Fragrant
b	Decanal	-	Sweet, Chemical/ Hydrocarbon, Waxy, Citrus, Floral
	2-methylundecanal	-	Fresh, Soft oriental, Chemical/ Hydrocarbon, Citrus, Sweet, Metallic, Waxy
	2-methyldecanal	-	Fresh, Dry, Citrus, Waxy, Water
c	Heptan-1-amine	-	Fishy/ Ammonia, Chemical/ Hydrocarbon
	Dibutylamine	-	Ammonia, Fishy/ Ammonia, Earthy/ Musty/ Moldy
d	Ethyl 2-(2-methyl-1,3-dioxolan-2-yl)acetate	-	Sweet, Fruity, Green, Woody
	2-tert-butylcyclohexyl acetate	-	Fruity, Woody, Green, Fragrant
	Ethyl butanoate	-	Sweet, Fruity
e	5,5-dimethyl-6-propyl-5,6-dihydro-2H-pyran-2-one	-	Minty
	6-(3-methylbutyl)-5,6-dihydro-2H-pyran-2-one	-	Dairy
	6-tert-butyl-6-methyl-5,6-dihydro-2H-pyran-2-one	-	Terpenes/ Pine/ Lemon, Minty
f	(4S)-1-methyl-4-(prop-1-en-2-yl)cyclohex-1-ene	(S)-(-)-limonene	Lemon

	(4R)-1-methyl-4-(prop-1-en-2-yl)cyclohex-1-ene	(R)-(+)-limonene	Citrus
g	Methyl (1R)-2,6,6-trimethylcyclohex-2-ene-1-carboxylate	-	Minty
	Methyl (1S)-2,6,6-trimethylcyclohex-2-ene-1-carboxylate	-	Fruity
h	(2R,5S)-5-methyl-2-(2-sulfanylpropan-2-yl)cyclohexan-1-one	-	Fruity
	(2S,5R)-5-methyl-2-(2-sulfanylpropan-2-yl)cyclohexan-1-one	-	Fuel/ Gas Station/ Solvent
i	(2R,5R)-5-methyl-2-(2-sulfanylpropan-2-yl)cyclohexan-1-one	-	Vegetables
	(2S,5S)-5-methyl-2-(2-sulfanylpropan-2-yl)cyclohexan-1-one	-	Fruity, Marshy/ Septic/ Sulphurous
j	(1S,2R,5S)-5-methyl-2-(propan-2-yl)cyclohexan-1-ol	D-menthol	Earthy/ Musty/ Moldy
	(1R,2S,5R)-5-methyl-2-(propan-2-yl)cyclohexan-1-ol	L-menthol	Minty
k	3-methylcyclopentadecan-1-one	-	Ammonia
	1-(4-tert-butyl-2,6-dimethyl-3,5-dinitrophenyl)ethan-1-one	-	Ammonia
	1-[1,1,2,6-tetramethyl-3-(propan-2-yl)-2,3-dihydro-1H-inden-5-yl]ethan-1-one	-	Ammonia
	2-[1-(3,3-dimethylcyclohexyl)ethoxy]-2-methylpropyl propanoate	-	Ammonia
